# Sodium Valproate, a Histone Deacetylase Inhibitor, Is Associated With Reduced Stroke Risk After Previous Ischemic Stroke or Transient Ischemic Attack

**DOI:** 10.1161/STROKEAHA.117.016674

**Published:** 2017-12-15

**Authors:** Rebecca L. Brookes, Siobhan Crichton, Charles D.A. Wolfe, Qilong Yi, Linxin Li, Graeme J. Hankey, Peter M. Rothwell, Hugh S. Markus

**Affiliations:** From the Stroke Research Group, Clinical Neurosciences, University of Cambridge, United Kingdom (R.L.B., H.S.M.); Division of Health and Social Care Research, Faculty of Life Sciences and Medicine, King’s College London, United Kingdom (S.C., C.D.A.W.); National Epidemiology and Surveillance, Canadian Blood Services, Ottawa, Ontario (Q.Y.); School of Medicine and Pharmacology, The University of Western Australia, Perth (G.J.H.); Department of Neurology, Sir Charles Gairdner Hospital, Perth, Australia (G.J.H.); Western Australian Neuroscience Research Institute, Perth (G.J.H.); and Stroke Prevention Research Unit, Nuffield Department of Clinical Neurosciences, University of Oxford, United Kingdom (L.L., P.M.R.).

**Keywords:** genetics, histone deacetylase, secondary prevention, sodium valproate, stroke, survival analysis

## Abstract

**Background and Purpose—:**

A variant in the histone deacetylase 9 (*HDAC9*) gene is associated with large artery stroke. Therefore, inhibiting HDAC9 might offer a novel secondary preventative treatment for ischemic stroke. The antiepileptic drug sodium valproate (SVA) is a nonspecific inhibitor of HDAC9. We tested whether SVA therapy given after ischemic stroke was associated with reduced recurrent stroke rate.

**Methods—:**

Data were pooled from 3 prospective studies recruiting patients with previous stroke or transient ischemic attack and long-term follow-up: the South London Stroke Register, The Vitamins to Prevent Stroke Study, and the Oxford Vascular Study. Patients receiving SVA were compared with patients who received antiepileptic drugs other than SVA using survival analysis and Cox Regression.

**Results—:**

A total of 11 949 patients with confirmed ischemic event were included. Recurrent stroke rate was lower in patient taking SVA (17 of 168) than other antiepileptic drugs (105 of 530; log-rank survival analysis *P*=0.002). On Cox regression, controlling for potential cofounders, SVA remained associated with reduced stroke (hazard ratio=0.44; 95% confidence interval: 0.3–0.7; *P*=0.002). A similar result was obtained when patients taking SVA were compared with all cases not taking SVA (Cox regression, hazard ratio=0.47; 95% confidence interval: 0.29–0.77; *P*=0.003).

**Conclusions—:**

These results suggest that exposure to SVA, an inhibitor of HDAC, may be associated with a lower recurrent stroke risk although we cannot exclude residual confounding in this study design. This supports the hypothesis that HDAC9 is important in the ischemic stroke pathogenesis and that its inhibition, by SVA or a more specific HDAC9 inhibitor, is worthy of evaluation as a treatment to prevent recurrent ischemic stroke.

The Wellcome Trust Case Control Consortium 2 genome-wide association study reported an association between a genetic variant on chromosome 7p21.1 and an increased risk of ischemic stroke because of large artery disease.^1^ The association was confined to large artery stroke and not present with cardioembolic or lacunar stroke. The association has been replicated in other cohorts of patients with stroke.^2,3^ The same genetic variant has also been associated with increased carotid intima–media thickness and asymptomatic carotid plaque,^4^ and less strongly, with coronary artery disease,^5^ suggesting an action via increasing atherosclerosis. The underlying gene is thought to be histone deacetylase 9 (*HDAC9* ).^4^ Mice with a deficiency of the *HDAC9* gene (*HDAC9^−/−^* apolipoprotein E–deficient) exhibit reduced aortic atherosclerosis compared with *HDAC9^+/+^* apolipoprotein E–deficient mice that do not have a deficiency.^6^ Furthermore, HDAC9 expression is upregulated in symptomatic carotid atherosclerotic plaques in man.^4^

The antiepileptic drug (AED) sodium valproate (SVA) is a nonspecific inhibitor of HDAC9 activity^7^ and has been shown to attenuate atherosclerosis in apolipoprotein E–deficient mice.^8^ A large Danish study suggested that although epilepsy was associated with an increased risk of incident stroke, the extent of this effect varied with the type of AED that was prescribed. SVA was associated with a decreased risk of both stroke and myocardial infarction compared with carbamazepine.^9,10^ A further large community study found a dose–response relationship with higher doses of SVA being associated with lower risks of incident stroke, but similar associations were also seen with some other AEDs, raising the possibility of survivor bias.^11^

These findings raise the hypothesis that inhibiting HDAC9 activity might offer a novel preventative treatment for large artery atherosclerotic ischemic stroke. We sought to indirectly test this hypothesis by exploring the association between exposure to SVA and subsequent risk of recurrent stroke in 3 large cohorts of patients with prior stroke or transient ischemic attack (TIA).

## Methods

### Data Sources

This project used data provided to us by 3 long-term follow-up stroke studies. Access to these separate data sources is therefore not available via this project.

Data were collected, and pooled, from 3 prospective studies recruiting patients with previous stroke or TIA and with long-term follow-up:

The SLSR (South London Stroke Register; n=4972) was a prospective population-based cohort study to record first-ever strokes in Lambeth and Southwark, London, United Kingdom.^12^ The final data set included data collected for patients with first-ever strokes between January 01, 1995, and September 30, 2014. Stroke diagnosis was confirmed by a study physician within 1 week of the event. Face-to-face follow-up took place at 3 months and then annually after the index event. For patients reaching at least 1 follow-up, the mean time from initial stroke to final follow-up was 4.6 years (SD=4.4; range=0–19);
The VITATOPS (Vitamins to Prevent Stroke Study; n=8164) was a clinical trial recruiting patients based on any stroke or TIA within the 7 months preceding randomization.^13^ Randomization took place between January 17, 1997, and December 29, 2008. Follow-up took place every 6 months from randomization to trial completion, either face-to-face or by telephone. For patients reaching at least 1 follow-up, the mean time from initial stroke or TIA to final follow-up was 3.4 years (SD=2.4; range=0–11);
The OXVASC (Oxford Vascular study; n=2113) is a population-based study of acute vascular events in Oxfordshire.^14,15^ The data set included here comprised all recruits ascertained between April 03, 2002, and March 31, 2012, with any first ischemic stroke or TIA in the study period. Multiple methods of follow-up were used, including face-to-face follow-up. Follow-up took place at 1, 6, 12, 24, 60, and 120 months. The mean time from initial stroke or TIA to final follow-up in this subset was 4.3 years (SD=3.4; range=0–12).

### Ethics

SLSR was approved by the following ethics committees: St Thomas’ Hospital, King’s College Hospital, Wandsworth, Riverside, and National Hospital for Neurology and Neurosurgery and the Institute of Neurology.

VITATOPS received ethics approval in the United Kingdom from the Multicentre Research Ethics Committee for Scotland, in New Zealand from the Multi-region Ethics Committee, and from local research ethics committees applicable to each participating center.

The Oxford Vascular Study was approved by the local research ethics committee (OREC A: 05/Q1604/70).

### Data Extracted

#### Study Populations

Study entry date was recorded as the date of index stroke or TIA. Classification of initial stroke pathology as ischemic or hemorrhagic was taken from the Oxfordshire Community Stroke Project (OSCP) or TOAST classifications (Trial of ORG 10172 in Acute Stroke Treatment) according to which classification had the least missing data per study. Where this was not available, the cases were categorized as unclassified.

#### Study End Point-Stroke Recurrence

Recurrent stroke occurrence and TOAST classification of recurrent ischemic stroke were collected across all studies. Stroke recurrence was captured at follow-up and subsequently checked by study physicians. These were then subtyped based on the TOAST classification.^16^ Data were censored if, without recurrence, the patient died, reached their final follow-up, or the study ended.

For the SLSR, stroke recurrence was defined as a new neurological deficit >24 hours after incident stroke and not considered to be because of edema, hemorrhagic transformation, or intercurrent illness. Recurrence within 21 days of the index stroke was only included if a different location was clearly indicated. For VITATOPS, recurrent stroke was defined as a new disturbance of focal neurological change lasting >24 hours or resulting in death and confirmed on imaging; all of the recorded recurrences were >24 hours from the index event. For OXVASC, recurrent stroke was recorded as any new neurological event lasting >24 hours or resulting in death and confirmed by a study physician who reviewed the surviving patients, the case records, and imaging.

#### Antiepileptic Treatment

Prescription data for antiepileptic medication were available for all 3 studies. Prescription data were recorded at baseline and then each follow-up. For OXVASC, AED data were available at baseline and 1-year follow-up. For the SLSR records of SVA, carbamazepine, phenobarbital, phenytoin, gabapentin, lamotrigine, levetiracetam, and occasional less frequently prescribed medications (other) were available. For VITATOPS records of SVA, carbamazepine, phenobarbital, and phenytoin were available. For OXVASC, records of SVA, carbamazepine, phenobarbital, phenytoin, gabapentin, and lamotrigine were available.

#### Other Variables

Other variables included in the analyses were study, age, sex, and diagnosis of epilepsy. Diagnoses of epilepsy were captured separately to antiepileptic medication. This was defined either as an existing diagnosis at baseline, made by a qualified physician, or a diagnosis recorded at follow-up. For VITATOPS, seizures were recorded as adverse events at each follow-up. These were classified as epileptic seizures where they met the criteria for the International League Against Epilepsy, and no baseline data were available on this. For the SLSR, baseline diagnoses were taken from patient medical records, and subsequent diagnoses were collected via self-report at follow-ups. For the OXVASC study, this included people with a history of >1 seizure in later childhood or adult life recorded at baseline by self-report.

### Statistical Analysis

**Figure 1.**
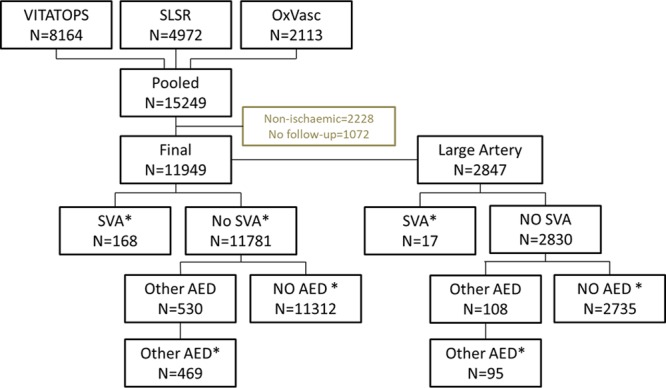
Flow cart of cases included in analysis. *Where prescription is before recurrent stroke or study end only. AED indicates antiepileptic drug; OXVASC, Oxford Vascular Study; SLSR, South London Stroke Register; SVA, sodium valproate; and VITATOPS, Vitamins to Prevent Stroke Study.

Figure 1 shows the selection of patients, exposure groups, and recurrent events. Patients were excluded from analyses if they had a hemorrhagic qualifying event or were lost to first follow-up.

The SVA exposure population was defined based on any prescription of SVA before recurrent stroke or study end. This was calculated based on any SVA-recorded exposure in the period preceding the study outcome, death, or final follow-up. We used the date of the first follow-up where there was a prescription of SVA to calculate this. SVA prescribed after recurrence was not considered SVA exposure.

Our protocol specified analysis was as follows: The SVA exposure populations were compared with 2 minimally selective control populations to avoid bias. (1) all other patients; this included everyone other than those receiving SVA; and (2) all other AED prescriptions: defined as any record of AED prescription at any time from study entry to final follow-up, including those with dates after recurrence but excluding those with concurrent prescriptions of SVA. Because the cohorts we used were from non-AED studies, the precise start date for AED use was not available and recorded instead as the first follow-up visit at which their use was recorded. For this reason, we initially included AED use at any time as our control population

It follows that our minimally selective criteria for the control populations could result in the other AED population, including some patients for whom AED prescriptions might be given after recurrent stroke. We, therefore, performed further secondary analyses with more restrictive comparison populations. We first compared patients receiving SVA with patients receiving no AEDs only, defined as patients without any AED prescriptions recorded before the study end or recurrent stroke. Second, we compared patients receiving SVA with patients only receiving other AED before stroke or study end based on the date of follow-up that these prescriptions were recorded.

Survival time was calculated as the number of days from the date of the index event (stroke or TIA) to the date of recurrent stroke occurrence or censoring. Life tables were calculated to describe the cumulative stroke-free survival for the exposure groups at 1, 5, 10, and 15 years. Survival curves were estimated with Kaplan–Meier and groups compared using the log-rank test in SPSS version 22. Cox regression was carried to calculate adjusted risk of a recurrent stroke. SVA exposure (SVA versus control), age, sex, history of epileptic symptoms, initial event type (TIA or stroke), and study were included in the model.

A secondary prespecified analysis was to determine whether any protective associations with SVA were confined to patients with large artery stroke, as might be expected from the genetic association data. Therefore, we repeated analyses in patients with large artery stroke.

Post hoc Cox regression analyses were performed for each study population individually. Exposure to SVA was compared with all patients without SVA exposure. Covariates were as above.

## Results

### Descriptive Data

The study cohort and patients included in the analysis are shown in the flow chart and in Table 1. A total of 11 949 patients were included in the pooled analyses, all of whom had a confirmed ischemic event at entry and follow-up available. The number in the SVA group was 168, and for those on other AEDs was 530. The total number of outcome events were 17 of 168 for patients prescribed SVA; 1470 of 11 781 for patients never prescribed SVA; 105 of 530 for patients prescribed other AEDs at any time; 1426 of 11 312 for patients not prescribed AEDs; and 44 of 469 for patients prescribed other AED when selected as prestroke/study end.

**Table 1.**
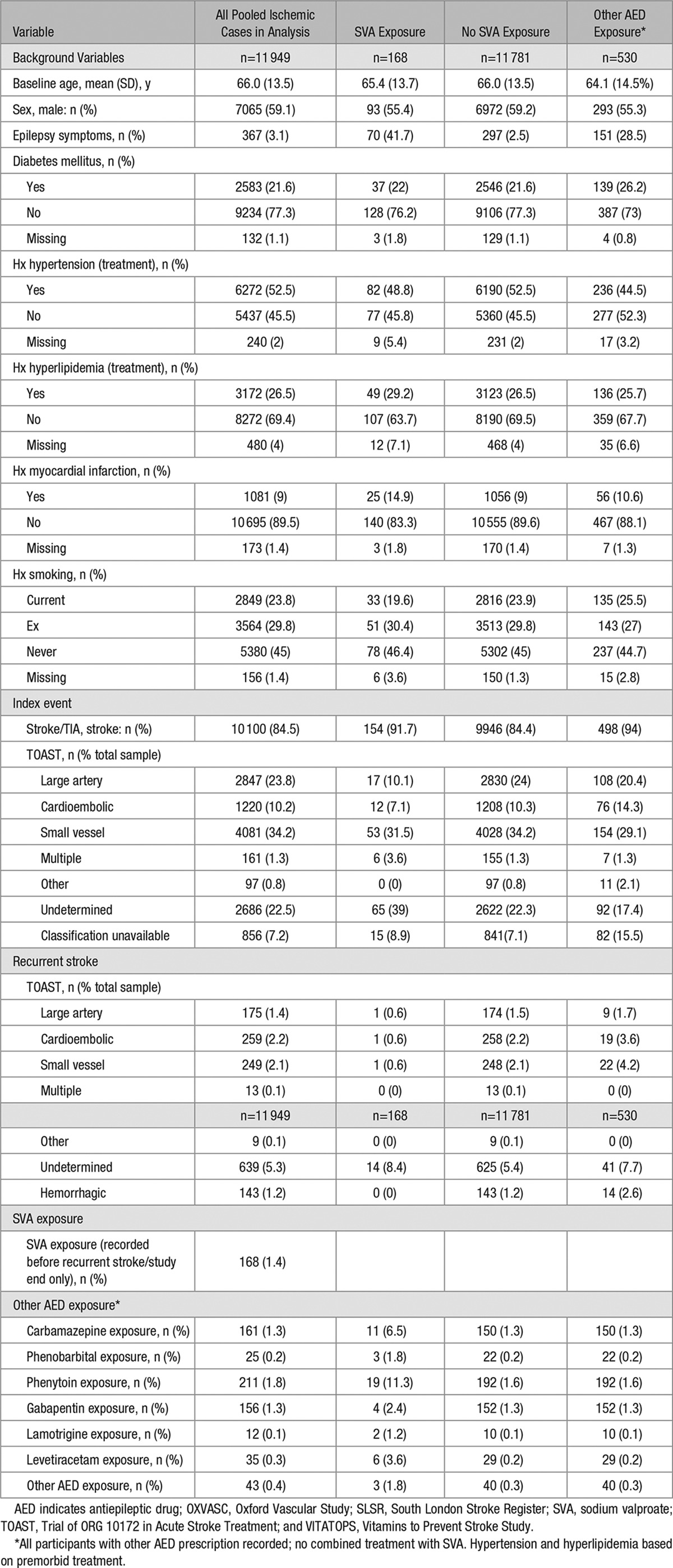
Pooled Background Data for OXVASC, VITATOPS, and SLSR

### Survival Models

The cumulative stroke-free survival, based on yearly data, was greater for the SVA group than for patients not prescribed SVA. Data are shown in Table 2.

**Table 2.**
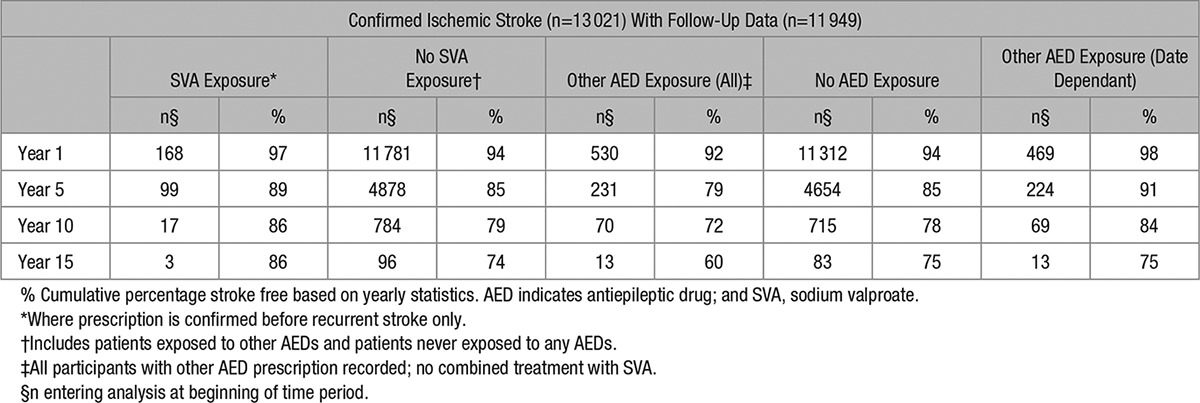
Cumulative Survival for All Entry Events (% Stroke Free)

**Figure 2.**
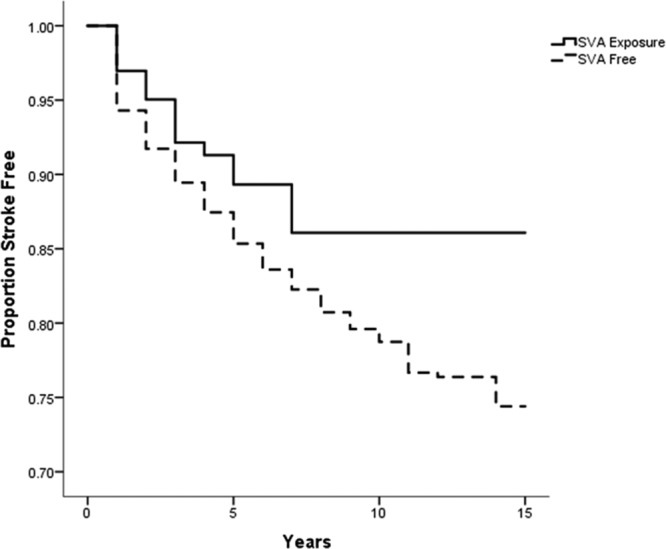
Survival curve comparing proportion of population free of recurrent stroke, in patients on sodium valproate (SVA) compared with those not on SVA.

**Figure 3.**
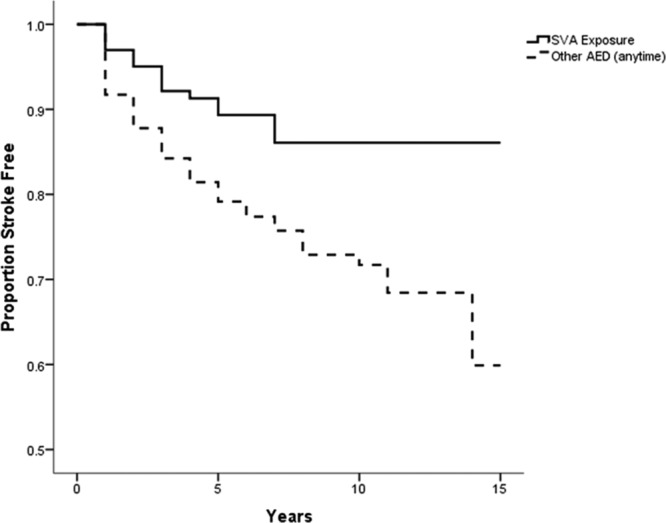
Survival curve comparing proportion of population free of recurrent stroke, in patients on sodium valproate (SVA) compared with those on other antiepileptic drug (AED).

For the nonselective control populations, Kaplan–Meier estimates were calculated for exposure to SVA, no exposure to SVA, and exposure to AED medication at any time. Log-rank tests showed that the difference between SVA exposure and no SVA exposure was not significant (χ^2^[1]=2.7; *P*=0.1) although there was a graphical trend indicating a difference in survival based on exposure at later time points; and the difference between SVA exposure and any other AED exposure was significant (χ^2^[1]=9.6; *P*=0.002). For the survival plots, see Figures 2 and 3.

For the selective control populations, Kaplan–Meier estimates were calculated for exposure to SVA, no exposure to AED medication, and exposure to any other AED medication prestroke/study end. A log-rank test showed that the difference between SVA exposure and no AED exposure was not significant (χ^2^[1]=2.91; *P*=0.088) although there was a graphical trend indicating a difference in survival at later time points, and the difference between SVA exposure and other AED exposure was not significant (χ^2^[1]=0.01; *P*=0.937).

### Hazard Models Adjusted for Covariates

Cox hazard models were calculated and adjusted for covariates. For the nonselective control comparisons, 2 models were created to account for the overlap in the control groups. Exposure to SVA was associated with a reduced risk of stroke compared with all patients without SVA exposure (hazard ratio [HR]=0.50; 95% confidence interval [CI], 0.31–0.82; Wald test, *P*=0.006). Exposure to SVA was associated with a reduced risk of stroke compared with the group prescribed other AEDs at any time (HR=0.41; 95% CI, 0.25–0.71; Wald test, *P*=0.001). For the selective control comparison, a single model was created. Group status was significant (Wald=27.9; *P*<0.0001). SVA was associated with a reduced risk of stroke compared with no AED exposure (HR=0.48; 95% CI, 0.35–0.67; Wald test, *P*<0.0001). Exposure to SVA was not associated with change in risk of stroke compared with other AED exposure group when selected as prestroke/study end (HR=1.2; 95% CI, 0.66–2.02; Wald test, *P*=0.62).

### Large Artery Stroke Analyses

Subgroup analyses were performed to determine whether any associations were specific to large artery stroke. For those patients with large artery index events, 17 patients were in the exposed to SVA group, 2830 were in the never exposed to SVA, of which 108 were exposed to AED medication other than SVA. Survival data are given in Table 3.

**Table 3.**
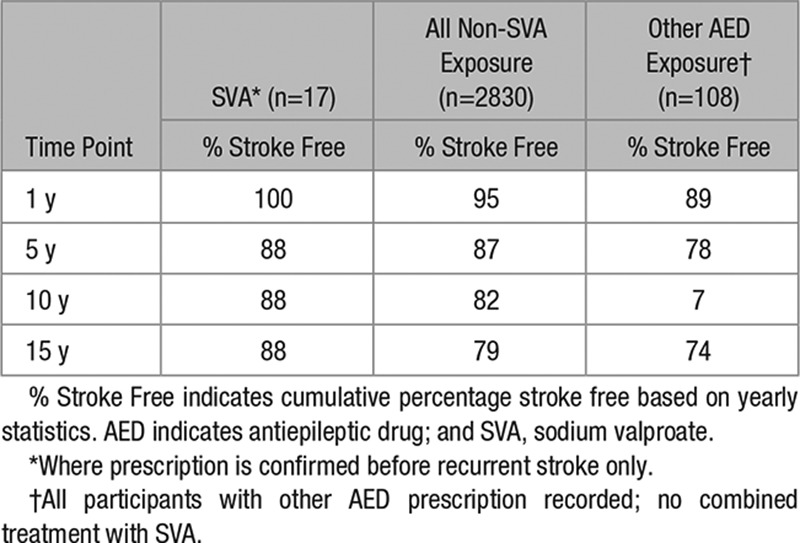
Cumulative Survival for Cases With Large Artery Entry Events

Kaplan–Meier estimates were computed. Although survival curves diverged for the groups, with a trend to better outcomes for those exposed to SVA, the data were limited by the small sample sizes. For a comparison between the SVA-exposed group and all other non-SVA–exposed patients, a log-rank test showed that the difference between the 2 survival curves was not significant (χ^2^[1]=0.073; *P*=0.787). For a comparison between the SVA-exposed group and the other AED group, a log-rank test showed that the difference between the 2 survival curves was not significant (χ^2^[1]=1.16; *P*=0.281).

### Post Hoc Analyses

Cox hazard models were calculated per study. Exposure to SVA was associated with a trend toward a reduced risk of stroke compared with all patients without SVA exposure for VITATOPS (HR=0.52; 95% CI, 0.26–1.0; Wald test, *P*=0.052) and the SLSR (HR=0.33; 95% CI, 0.12–0.92; Wald test, *P*=0.033) but not OXVASC (HR=1.2; 95% CI, 0.40–3.4; Wald test, *P*=0.781).

## Discussion

This analysis of 3 large cohorts of patients with prior stroke or TIA was undertaken to explore an a priori hypothesis (ie, the hypothesis was generated from published studies^1–11^ before the epidemiological data were analyzed) that exposure to an inhibitor of HDAC, in the form of SVA, may be associated with a lower risk of recurrent stroke compared with nonexposure or to exposure to other AEDs. Although the design of our study is prone to systematic and random error and cannot infer causality, the results provide some evidence for the prestudy hypothesis and suggest that SVA, a nonspecific HDAC inhibitor, may be associated with a reduced stroke recurrence rate.

Previously, data have suggested that SVA reduces stroke risk in a stroke-free population, but this analysis provides new data suggesting that such an effect can also be found in patients who have already presented with ischemic stroke. This is particularly relevant if HDAC9 inhibition is to be considered as a potential secondary preventative treatment for stroke.

The results are broadly consistent with the results of 2 large population-based studies in Denmark and the United Kingdom. In a Danish study, SVA was associated with a lower risk of both myocardial infarction and stroke when compared with other AEDs.^9,10^ In the British study, which used the Clinical Practice Research Database, SVA exposure was associated with a reduced risk of myocardial infarction but not ischemic stroke.^11^ However, when a dose–response analysis was performed, longer exposure to SVA was associated with a reduced risk of ischemic stroke although similar associations were found with other AEDs raising questions about the specificity of the association.

In our study when comparing SVA use with all other patients with ischemic stroke and those patients with ischemic stroke on no AED, there was a highly significant reduced risk of stroke. This was replicated in our preplanned analysis comparing patients with SVA with those taking any other AED. Because of the use of cohorts collected for other study purposes, we did not have a precise start date for AEDs, and we therefore used the date of the follow-up at which the medication was recorded. For this reason, we initially included AED use at any time as our control population. However, when we performed further exploratory analyses in the data where the AED record dates were before recurrent stroke, the difference was no longer significant. This raises the possibility that the significant risk reduction seen in the SVA group compared with the 3 control groups might be because of some difference if patient characteristics between the 2 groups. Such bias are impossible to exclude in a cohort study, such as this, and confirming whether SVA does indeed reduce stroke risk will require a randomized trial design. One potential bias might be if stroke subtype differs in patients on SVA, for example, lacunar stroke might have a lower risk of epilepsy requiring AED therapy and a lower risk of stroke recurrence. However, there was no evidence of any difference in lacunar stroke frequency between groups (Table 1). A second possible issue is related to the collection of prescription dates which were based on follow-up rather than prescription date. Alternatively, it may be a possibility that other AEDs also have some inhibitory effect on HDAC9 although evidence for this is less clear. Notwithstanding, SVA exposure appeared overall to have a positive effect compared with no SVA exposure.

Strengths of this study include the prospective design, standardized diagnostic criteria for the qualifying TIA and stroke, and prolonged follow-up. Two of the 3 cohorts were population-based studies, reducing the risk of any selection bias. The third was a large randomized clinical trial that again incorporated prolonged follow-up.

However, the data sets also had some limitations. When examined separately as a post hoc analysis, 2 of the data sets yielded the same trend toward and effect for SVA, OXVASC did not. However, OXVASC had a small number of patients with confirmed SVA exposure (n=11), making it difficult to interpret in isolation. Sufficient data were not available to assess dose–response relationships, and therefore analyses were restricted to SVA prescribed at any time during follow-up. Because of the nature of data collection, often at annual follow-up, determining the exact start date of AED was not possible, and these were recorded as starting at the time the patient was first followed-up on that AED. Despite the large sample size of the >10 000 patients with stroke, the number of participants taking SVA and other AEDs was relatively few (in the hundreds) which limited statistical power. Furthermore, the number of cases of large artery stroke was too small to reliably test whether any protective effect was specific to this subtype, as hypothesized from the genetic association data.^1^ It is also inherent in this type of data that the outcomes are more heavily weighted in the early years (because of early recurrence and long follow-up), but this is consistently the case across comparison groups. It may be that data sets with more power would benefit for an analysis splitting short- and long-term outcomes in relation to AED medication.

It is also acknowledged that SVA is a nonspecific HDAC inhibitor (inhibiting a wide range of HDACs) and has other actions, independent of HDAC9 inhibition. Hence, a more specific inhibitor of HDAC9 might have a stronger effect in reducing the risk of recurrent stroke. HDACs are a class of enzymes that remove acetyl groups from an ε-N-acetyl lysine amino acid on a histone, allowing the histones to wrap the DNA more tightly. There are 18 HDACs in humans. Eleven of the HDACs are zinc dependent, classified on the basis of homology to yeast HDACs: class I includes HDACs 1, 2, 3, and 8; class IIA includes HDACs 4, 5, 7, and 9; class IIB, HDACs 6 and 10; and class IV, HDAC11.^17^

This study provides some support for the hypothesis that HDAC9 is important in the pathogenesis of ischemic stroke and that its inhibition, by SVA or a more specific HDAC9 inhibitor, is worthy of evaluation as a treatment to prevent recurrent ischemic stroke. However, because of limitations in a cohort study of this design, and possible unidentified bias, determining whether HDAC9 inhibition does reduce stroke risk requires randomized controlled trials of SVA or other HDA9 inhibitors.

## Acknowledgments

We thank Adina Feldman for assistance in data management and analysis. Dr Markus is supported by a National Institute for Health Research (NIHR) Senior Investigator award and the NIHR Biomedical Research Centre at Cambridge. Dr Rothwell is in receipt of an NIHR Senior Investigator Award and a Wellcome Trust Senior Investigator Award. Dr Wolfe is supported by the NIHR Biomedical Research Centre at Guy’s and St Thomas’ National Health Service Foundation Trust and King’s College London. The views expressed are those of the author(s) and not necessarily those of the National Health Service, the National Institutes of Health Research, or the Department of Health.

## Sources of Funding

This project was funded by a British Heart Foundation (PG/13/30005). The VITATOPS trial (Vitamins to Prevent Stroke Study) was funded by the National Health and Medical Research Council of Australia Project Grants 110267 (2000–2004) and 403913 (2006–2008) and Program Grants 251525 (2003–2007) and 454417 (2007–2011). The Oxford Vascular Study has been funded by Wellcome Trust, Wolfson Foundation, UK Stroke Association, British Heart Foundation, and NIHR Oxford Biomedical Research Centre and supported by the facilities of the Acute Vascular Imaging Centre, Oxford.

## Disclosures

Drs Markus and Wolfe received funding from the British Heart Foundation as above to fund this project. The other authors report no conflicts.

## Supplementary Material
