# Supplementary material for: Sodium Valproate, a Histone Deacetylase Inhibitor, Is Associated With Reduced Stroke Risk After Previous Ischemic Stroke or Transient Ischemic Attack
Source: Stroke. 2017 Dec 15;49(1):54–61. doi: 10.1161/STROKEAHA.117.016674 (PMC5753817; doi:10.1161/STROKEAHA.117.016674)
Supplement: Supplementary file 1 [file str-49-054-s001.pdf]

American Heart Association | American Stroke Association®

Acknowledgment Permission Form

Journal Stroke
Manuscript Number STROKE/2017/016674R3
First Author Rebecca L Brookes
Title of Work Sodium valproate, an HDAC inhibitor, is associated with reduced stroke risk after previous ischaemic stroke or TIA

Authors must provide written permission/approval from all individuals mentioned by name in the Acknowledgments section of a submitted manuscript. By signing this form, any and all acknowledged persons therefore state that they have read and approved the mention of their names in the Acknowledgment section of the aforementioned paper.

Name (1) ADINA FELDMAN	Signature Adina L Feldman	Date 11/10/2017
Name (2)	Signature	Date
Name (3)	Signature	Date
Name (4)	Signature	Date
Name (5)	Signature	Date
Name (6)	Signature	Date
Name (7)	Signature	Date
Name (8)	Signature	Date
Name (9)	Signature	Date
Name (10)	Signature	Date
Name (11)	Signature	Date
Name (12)	Signature	Date
Name (13)	Signature	Date
Name (14)	Signature	Date
Name (15)	Signature	Date
Name (16)	Signature	Date
Name (17)	Signature	Date
Name (18)	Signature	Date
Name (19)	Signature	Date
Name (20)	Signature	Date
